# Identification of the *Plasmodium berghei* resistance locus 9 linked to survival on chromosome 9

**DOI:** 10.1186/1475-2875-12-316

**Published:** 2013-09-11

**Authors:** Selina ER Bopp, Evelyn Rodrigo, Gonzalo E González-Páez, Mary Frazer, S Whitney Barnes, Clarissa Valim, James Watson, John R Walker, Christian Schmedt, Elizabeth A Winzeler

**Affiliations:** 1 Department of Pediatrics, University of California, San Diego, School of Medicine, La Jolla, CA, USA; 2 The Genomics Institute of the Novartis Research Foundation, La Jolla, CA, USA; 3 The Scripps Research Institute, La Jolla, CA, USA; 4 Present Address: Harvard School of Public Health, Boston, MA, USA

**Keywords:** *Plasmodium berghei*, Experimental cerebral malaria, Quantitative trait locus, Malaria, Basophil, Chromosome mapping, Mouse

## Abstract

**Background:**

One of the main causes of mortality from severe malaria in *Plasmodium falciparum* infections is cerebral malaria (CM). An important host genetic component determines the susceptibility of an individual to develop CM or to clear the infection and become semi-immune. As such, the identification of genetic loci associated with susceptibility or resistance may serve to modulate disease severity.

**Methodology:**

The *Plasmodium berghei* mouse model for experimental cerebral malaria (ECM) reproduces several disease symptoms seen in human CM, and two different phenotypes, a susceptible (FVB/NJ) and a resistant mouse strain (DBA/2J), were examined.

**Results:**

FVB/NJ mice died from infection within ten days, whereas DBA/2J mice showed a gender bias: males survived on average nineteen days and females either died early with signs of ECM or survived for up to three weeks. A comparison of brain pathology between FVB/NJ and DBA/2J showed no major differences with regard to brain haemorrhages or the number of parasites and CD3+ cells in the microvasculature. However, significant differences were found in the peripheral blood of infected mice: For example resistant DBA/2J mice had significantly higher numbers of circulating basophils than did FVB/NJ mice on day seven. Analysis of the F2 offspring from a cross of DBA/2J and FVB/NJ mice mapped the genetic locus of the underlying survival trait to chromosome 9 with a Lod score of 4.9. This locus overlaps with two previously identified resistance loci (*char1* and *pymr*) from a blood stage malaria model.

**Conclusions:**

Survival best distinguishes malaria infections between FVB/NJ and DBA/2J mice. The importance of *char1* and *pymr* on chromosome 9 in malaria resistance to *P. berghei* was confirmed. In addition there was an association of basophil numbers with survival.

## Background

The majority of infectious disease deaths worldwide are due to malaria, HIV and tuberculosis and malaria is the most common parasitic disease in the world. Five malaria parasite species can cause malaria in humans with *Plasmodium falciparum* being responsible for the most deaths that result from the severe form of disease. Severe malaria is characterized by anaemia, respiratory distress, and cerebral malaria (CM). Many factors contribute to the manifestation of CM. Cytoadherence of infected red blood cells (RBCs) in brain micro-vessels and other organs; parasite products such as toxins and possibly haemozoin; local and systemic production of cytokines and chemokines by the host; the activation, recruitment and infiltration of inflammatory cells are also involved in the development of CM and the neurological symptoms [1]. Although parasite sequestration, haemorrhage and inflammation are often found in brains of CM patients, CM is not a homogenous syndrome. Variations in the clinical features of CM may be due to genetic differences in the host or the parasite, the immune response of the patient and/or environmental factors.

For ethical and logistical reasons CM can only be studied using brains from fatal cases (not during the course of an infection or after successful treatment). Primate models of CM, such as *Plasmodium knowlesi* and *Plasmodium coatneyi* infections in Rhesus monkeys [2,3] and *P. falciparum* infections in squirrel monkeys [4] exist; however, these are expensive and use of non-human primates are problematic. The *Plasmodium berghei* ANKA mouse model of experimental cerebral malaria (ECM) replicates most of the human CM symptoms and is the most commonly used model for CM [5,6]. Susceptible mouse strains such as CBA and C57BL/6 develop ECM with ataxia, paralysis, and coma [7]. Blood brain barrier disruption and vascular leakage are also observed in mice with ECM [8,9] as well as accumulation of platelets [10,11], monocytes and macrophages in the micro-vessels [12,13]. Other mouse strains do not show symptoms of ECM [14,15].

It is now an established fact that the genetic background of the host can influence the outcome of disease. For example, coevolution of the parasite and the host has led to an increase of beneficial alleles in malaria endemic areas. These include sickle cell trait (HbAS) and haemoglobinopathies such as thalassaemias and glucose-6-phosphate dehydrogenase deficiency as well as a number of immune-modulating genes that have been associated with resistance or susceptibility to *P. falciparum* malaria in humans (reviewed in [16]). Linkage and gene association studies in humans are hampered by the need for large number cases and controls. Genome-wide analysis in inbred mouse strains eliminates genetic variability between individuals and serves as a model to study resistance and susceptibility to *Plasmodium* in a well-defined system. To date, ten genetic loci that contribute to the control of parasitaemia have been identified in *Plasmodium chabaudi* infections (*char1-11*) [17-25]. Similarly, seven genetic loci associated with resistance to ECM (*berr1, berr2, berr5, berr6, berr7, cmsc* and a locus on chromosome 18) [26-31] and one locus associated with reduced liver infection (*belr1*) have been mapped using the *P. berghei* model [32]. An additional locus (*berr3*) was identified that controlled resistance to lethal infection and one locus that enhanced survival time (*berr4*) [29]. A combinatorial effect of loci *berr1* and *berr3* was suggested to be responsible for the clearance of parasites and survival [29,33].

In a previous study, 32 different mouse strains were characterized for survival, body temperature and parasite distribution in organs. Survival was mapped to a sixth berghei resistance locus (*berr6*) containing the peroxisome proliferator-activated receptor gamma (*Pparg*) using haplotype associated mapping (HAM) [15]. In this study, a susceptible (FVB/NJ) and a resistant (DBA/2J) mouse strain were characterized in more detail to identify early predictors of disease. Survival was the most robust resistance trait and a whole genome scan identified chromosome 9 as a key regulator for survival in a F2 cross. Genes in the locus identified in this study might be potential targets for therapeutic interventions.

## Methods

### Ethic statement

All animal experiments were approved by the Institutional Animal Care and Use Committee (IACUC) and conducted in agreement with the NIH policy.

### Mice

The DBA/2J and FVB/NJ inbred strains were purchased from The Jackson Laboratories (JAX). Eight to twenty week-old mice were used in the study. Mice were housed in a pathogen free facility at the Genomics Institute of the Novartis Research Foundation (GNF) and all experiments were approved by the IACUC and conducted in agreement with the NIH policy.

### Genotyping

Before infection, tail biopsies were obtained from all F2 animals and genomic DNA was isolated by a standard procedure involving proteinase-K treatment [34]. Mice were genotyped for a total of 355 SNPs and microsatellite markers. SNP genotyping was performed at GNF by using the Sequenom MassARRAY system and a custom panel of SNPs distributed across the genome [35].

### Infection and parasites

*Plasmodium berghei* ANKA strain *PbGFP–LUC*_*SCH*_[36] was used for all infections. Parasites from frozen stocks of this strain were propagated and maintained in donor mice. Mice were infected intraperitoneally with 1×10^6^-parasitized red blood cells obtained from a donor mouse. Parasites were preserved in Alsever’s solution containing 10% glycerol and stored in liquid nitrogen.

### Parasitaemia

Parasite levels in the blood were monitored by flow cytometry analysis. Blood was obtained from the tip of the tail: the end of the tail was clipped with a clean razor and ~5 μl of whole blood was fixed in 1 ml of 0.25% glutaraldehyde in PBS (pH 7.4) and stored at 4°C before being stained. For subsequent bleeds the scab was removed. Fixed blood was incubated for 1 h at 37°C in the dark with 1 μM Hoechst 33258 in PBS. The samples were analysed by cytofluorometry using a FACStar plus cytofluorometer (Becton Dickinson, CA, USA) equipped with a Coherent Innova 90 laser tuned to UV excitation (351 nm, 200 mW). A 424DF44 filter was selected as the emission filter for the blue Hoechst fluorescence. Files were analysed by Cellquest 3.2 software. Erythrocytes were gated by forward and side scatter and subsequently infected erythrocytes were selected by gating for Hoechst fluorescence. For each sample, 3,000 events were acquired and recorded. The percentage of infected RBCs was determined on the basis of the positive blue fluorescence of infected erythrocytes.

### ECM scores

Mice were monitored twice a day, starting three days post infection, and clinical ECM evaluated. The score recorded later during the day was used for the ECM progression per day when different from the earlier score of the same day. Clinical ECM scores were defined by the presentation of the following signs: ruffled fur, hunching, wobbly gait, limb paralysis, convulsions, and coma. Each sign was given a score of 1. Additionally, a score of 0.5 was given if the mice were unresponsive to touch and too weak to stand without paralysis or convulsions. Animals with severe ECM (accumulative scores > 3.5) were sacrificed by isoflurane asphyxiation according to ethics guidelines, and survival was deemed to be the same day due to the rapid progress of the disease.

### Evans blue

200 μl 1% Evans blue dye were injected into each mouse via the tail vein. After 1 h the mouse was euthanized with isoflurane, and subsequently the brain was removed and placed in 1 ml formalin for 48 h at room temperature to extract the Evans blue dye. The optical density of the extracted dye was measured with a SPECTRAmax PLUS ROM v3.13 fluorescence plate reader in the absorbance mode at 620 nM. The absorbance for the extracted dye from an infected brain was normalized to the dye extracted from an uninfected control brain prepared on the same day.

### Complete blood count

Mice were lightly anesthetized with isoflurane and 70 μl blood was collected from the eye by retro-orbital bleeding. Blood was diluted 1:3 in CELL-DYN solution and mixed for 10 min on a tube-roller and subsequently analysed in a CELL-DYN 3700 multi-parameter haematology analyzer (Abbott, IL, USA) according to the manufacturer’s instructions. Since control mice did not show significant differences between day two and seven by paired student t test their values were pooled.

### Histology

After dissection, the brains were bisected sagittally along the midline, half were fixed in 10% formalin then embedded in paraffin, and the other half were fixed in 4% paraformaldehyde, embedded in Tissue-Tek OCT (Sakura Finetek USA, Inc., CA, USA) and then frozen. Paraffin embedded brains were serially sectioned in the sagittal plane at 5 microns. Every 10th slide was stained with haematoxylin-eosin (H&E) and the adjacent slide was stained with Giemsa. Sections were also stained with anti-CD3 and anti-GFP antibodies on a Ventana Staining platform. After deparaffinization, heat induced antigen retrieval, avidin/biotin blocking, and serum blocking the slides were incubated with the following antibodies: CD3 (Cat#A0452, Dako, CA, USA) and GFP (Cat# LS-C67081, LifeSpan BioSciences, Inc., WA, USA). Species appropriate biotinylated secondary antibody was applied and 3,3′ diaminobenzidine (DAB) chromogen detection was used.

### Statistical analysis

Quantitative trait locus was performed using the R/QTL software [37]. This software calculates the logarithm of odds (Lod) scores over intervals between linked markers, representing the likelihood of genetic linkage of quantitative phenotypes with markers along the chromosome. The hidden Markov methodology technology was used to calculate the probabilities of the true underlying genotypes given the observed multipoint marker data, with possible allowance for genotyping errors. The Viterbi algorithm filled in missing genotypes and subsequently marker regression analysis was performed. The level of statistical significance was empirically determined by permutation tests (1,000). Markers and mouse IDs for which more than 80% of the samples showed no good signal were not considered. Statistical significance of differences in survival between FVB/NJ and DBA/2J mice were assessed through partial likelihood ratio tests estimated via Cox proportional-hazards models. Since those differences in survival could not only be confounded by strain, but also vary according to mouse gender, in Cox models, results were adjusted by gender and compared survival across genders by testing interaction terms between gender and strain. The same approach was used to compare survival of male and female F2 mice.

Comparisons of changes over time in body weight, parasitaemia (natural log transformed percent parasitaemia with a constant), and ECM between FVB/NJ and DBA/2J were done in linear regression models with a random intercept to account for within mice correlation across days. These models tested whether slopes of linear trends of FVB/NJ and DBA/2J were statistically significantly different (through inclusion of an interaction term between strain and day) before and after accounting for the effect of gender at day 0 (baseline). Differences in the trends were also evaluated over time between FVB/NJ and DBA/2J mice for both genders through inclusion of interaction terms. Model selection was based on F-tests.

A student’s t test was used to check strain specific statistically significant differences for parasitaemia and body weight on specific days. A Mann-Whiney-Wilcoxon Rank Sum test was used to calculate daily differences in ECM scores. Differences between control mice and infected mice regarding haematocrit, blood composition and absorbance of Evan’s blue were assessed by one-way ANOVA analysis with a Dunnett’s post-hoc test.

In all tests, p-values were considered statistically significant if ≤ 0.05, except when studying interactions with gender in which p-values ≤ 0.10 were considered borderline significant and yield reporting of gender specific associations. Statistically significant differences were calculated in GraphPad Prism and Splus 8.0.

## Results

### Phenotypic differences between DBA/2J and FVB/NJ mice

To identify the gene regions responsible for susceptibility or resistance to ECM caused by *P. berghei* infection, the phenotype that best distinguishes a susceptible and a resistant mouse strain or that allows early prediction of severity of disease was assessed. To date only C57BL/6 mice have been used in combination with a resistant strain in *P. berghei* linkage studies. However since another susceptible mouse strain might give new insight into the disease, a susceptible FVB/NJ and a resistant DBA/2J mouse strains were used for this analysis [15].

**Figure 1:**
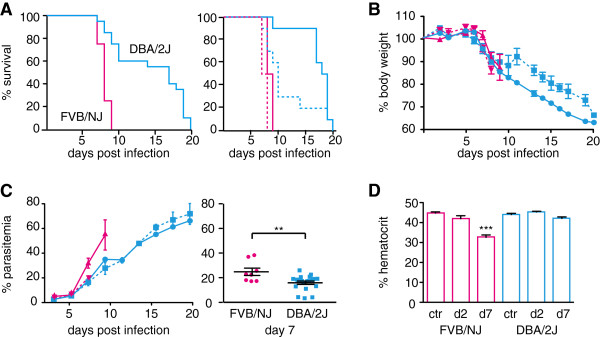
**Phenotypic analysis of DBA/2J and FVB/NJ mice.** Ten male and ten female DBA/2J (blue) and four male and four female FVB/NJ (pink) mice were infected with *P. berghei*. Mice were monitored twice daily. **A**. Survival for total FVB/NJ and DBA/2J mice, the right graph shows the survival curves for the female (dotted line) and the male mice (solid lines) separately. **B**. The loss of body weight over the course of infection. **C**. Parasitaemia was measured every second day starting on day three post infection. The graph on the right shows parasitaemia for FVB/NJ and DBA/2J mice on day seven. **D**. Haematocrit measured in control mice (ctr) or in infected mice on day two and seven post infection (d2, d7). Shown are the averages with the standard error. Stars indicate statistical significance by unpaired Student’s t test **(A, C)** or one-way ANOVA with Dunnett’s post-hoc test compared to the control **(D)**, ** p > 0.01, *** p > 0.001. Statistical differences regarding survival curves and progress of parasitaemia and body weight are discussed in the text.

Since body temperature and parasite load in organs were not as informative as survival in earlier studies [15], additional phenotypes such as signs of ECM, parasitaemia, body weight and haematocrit were included in this study. In previous studies DBA/2J mice presented a resolving phenotype of ECM where mice showed signs of ECM early during infection but recovered and died of high parasitaemia late during infection [38]. Therefore, ten male and ten female DBA/2J mice and four male and four female FVB/NJ mice were infected. All FVB/NJ mice died within nine days of infection, and differences in the rate of death of DBA/2J and FVB/NJ mice were statistically significantly different before (p < 0.001) and after accounting for the effect of gender (p < 0.001). Overall, there was no significant difference in survival between males and females (p = 0.143). Surprisingly, DBA/2J mice did not show a resolving phenotype of ECM: seven female and one male DBA/2J died before day eleven and the remaining DBA/2J survived for thirteen days or longer (Figure 1A). As a consequence the DBA/2J mice were put into two groups: in the susceptible group mice died before day thirteen and in the resistant group mice survived for thirteen days and more. Only three females were resistant in contrast to nine males, however survival in DBA/2J females was not statistically significantly different from survival in DBA/2J mice (p = 0.15).

Loss of body weight is a general feature of most mouse malaria infections [39]. To determine whether weight loss was a valid predictor of disease severity or survival, mice were weighed daily. Both strains showed a significant drop in body weight from day seven onwards, regardless of disease symptoms (FVB/NJ p = 0.0068 and DBA/2J p = 0.0001, Figure 1B). Changes in body weight over time from day five on were significantly different between FVB/NJ and DBA/2J mice (p = 0.01). This most likely resulted from a significant difference in changes in body weight over time between DBA/2J and FVB/NJ females (p = 0.01) rather than males (p = 0.27) (p interaction term = 0.03). The weight of DBA/2J mice decreased to below 70% of their initial weight. Total weight loss correlated with survival in DBA/2J mice but the onset of loss in body weight was similar for both strains and only different for DBA/2J females. Therefore weight loss was not used as a predictor of severity of disease.

Parasitaemia was measured every second day starting on day three post infection. As expected parasitaemia increased over the course of infection and there was a positive correlation between survival and terminal parasitaemia in both strains (FVB/NJ: r = 0.751, p = 0.0317, DBA/2J: r = 0.7153, p = 0.0004, Figure 1C). Changes in parasitaemia over time from day five on between strains were statistically significant (p before and after adjustments by gender < 0.0001) and results were not affected by gender (p = 0.80); comparison between genders was borderline significant (p = 0.06). The parasitaemia was significantly higher in FVB/NJ mice on day seven than in DBA/2J mice (p = 0.0045) suggesting that initially DBA/2J mice control parasitaemia better than FVB/NJ mice. Nevertheless, DBA/2J mice are not able to control parasitaemia and hyper parasitaemia is a likely cause of death for the mice that survived for more than thirteen days.

Anaemia is a major symptom of severe disease in humans and mice. The haematocrit is a direct way to measure anaemia. To measure the onset of anaemia the haematocrit was measured on day two and day seven of the infection. There was a significant decrease in the haematocrit of FVB/NJ mice on day seven compared to the uninfected control; however, there was no significant decrease in DBA/2J mice, indicating a genetic difference between the two strains (Figure 1D). There were no significant gender specific differences within or between strains. No correlation between the haematocrit and the parasitaemia on day seven was observed indicating that haemolysis of infected red blood cells was probably not responsible for the drop in haematocrit. Surprisingly, survival in DBA/2J mice was negatively correlated with haematocrit on day seven (r = -0.5046, p = 0.0327). As such this implies that anaemia was not the cause of death for the eight DBA/2J mice that died early.

Out of all assessed phenotypes only loss of body weight was affected by gender and gender specific genetic factors were therefore not operating to generate phenotypic differences.

### Haemorrhage in the brain is insufficient to induce ECM

**Figure 2:**
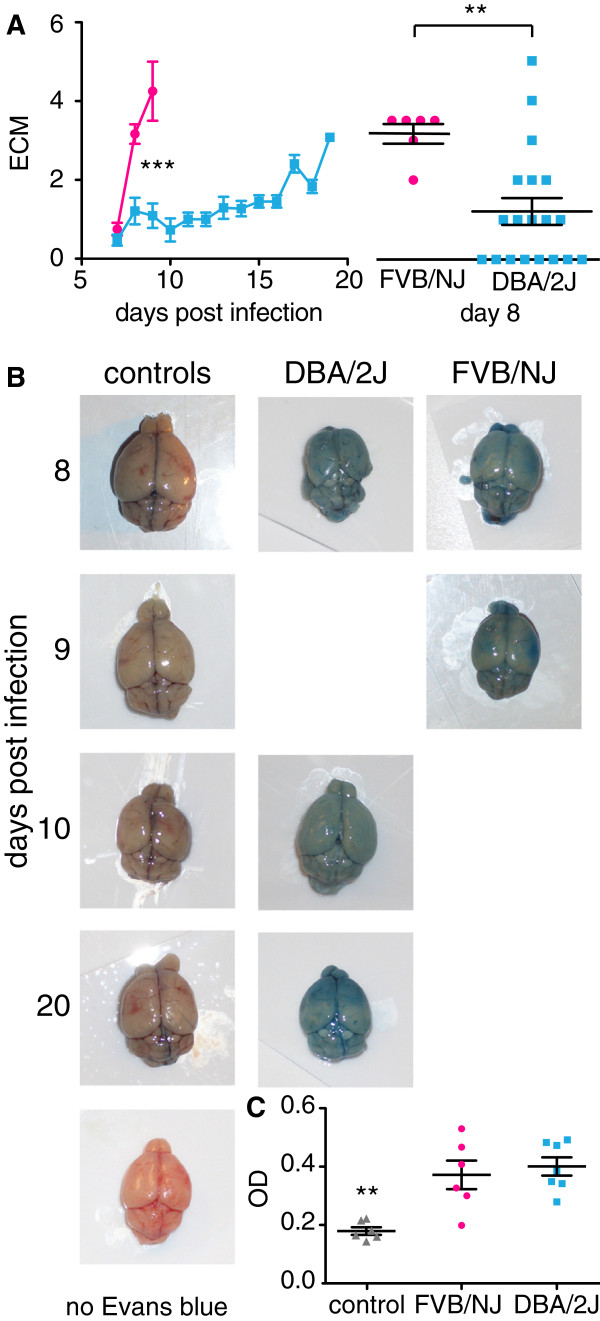
**Experimental cerebral malaria in FVB/NJ and DBA/2J mice.** Mice were scored twice daily for symptoms of ECM. **A**. Average ECM scores over the course of infection with standard errors. The inset shows statistically significant differences in ECM scores on day eight (Linear regression model, *** p < 0.001 and Mann-Whiney-Wilcoxon Rank Sum test, ** p > 0.01). **B**. Mice with ECM scores of 3.5 or higher were injected with Evans blue 60 minutes prior to dissection. Representative images of brains from infected mice and uninfected control mice are shown. The days post infections are indicated on the left. The bottom control shows a brain without Evans blue injection prior to dissection. **C**. Evans blue was extracted from the brains and the absorption was measured. Shown are the averages with the standard error (one-way ANOVA with Dunnett’s post-hoc test compared to the control, ** p > 0.01).

When infected with *P. berghei* ANKA parasites, certain mouse strains such as C57BL/6 and CBA, develop symptoms of ECM including ataxia, fitting, respiratory distress and coma [7]. The conditions worsen quickly and oftentimes death occurs within four to five hours of the onset of neurological signs. Disruption of the blood brain barrier and vascular leakage have been observed in brains of mice showing signs of ECM. To determine whether FVB/NJ or DBA/2J mice succumb to ECM, the mice were examined twice daily and symptoms of ECM were scored as following: 1: ruffled fur, 2: hunching, 3: wobbly gate, 3.5 no response to touch, 4: limb paralysis, 5: seizure and 6: coma. ECM scores increased rapidly in the FVB/NJ mice, moderately in the susceptible DBA/2J mice and slowly in the resistant DBA/2J mice. This explains the initial increase followed by a drop in ECM scores in the DBA/2J mice (Figure 2A). There were significant differences in ECM trends over time between the strains (p before and after accounting for the baseline effect of gender < 0.001) but those differences were comparable in males and females (p = 0.45). All FVB/NJ mice had scores of 2 and more on day eight in comparison to 30% of the DBA/2J mice. Only one FVB/NJ mouse and two DBA/2J mice were either paralyzed or had seizures. None of the DBA/2J mice that survived for over thirteen days had scores higher than 3.5 indicating that only those mice that died early had severe signs of ECM.

Since only a small fraction of the mice showed strong signs of ECM, the extent of ECM was further investigated in these strains. The degree of vascular leakage in brains of FVB/NJ and DBA/2J mice was investigated with Evans blue. Mice that had ECM scores of 3.5 and higher were injected with Evans blue into the tail vein and the absorption of Evans blue in the brain was measured (Figure 2B). The blood vessels of brains from control mice were red indicating that the blood–brain barrier had not been disrupted and Evans blue did not cross the blood–brain barrier. In contrast, blood vessels of mice infected with *P. berghei* were stained blue and infected mice had significantly higher absorption of Evans blue than the uninfected control mice. All of the brains from infected mice were stained blue and there was no significant difference of Evans blue absorption between FVB/NJ and DBA/2J mice and no correlation of the amount of leakage of Evans blue into the brain with survival or ECM scores. While only three mice showed strong signs of ECM such as paralysis or seizures, leakage of blood into the brain was observed in all thirteen mice confirming that high ECM scores are associated with break down of the blood brain barrier.

### Accumulation of parasites and T cells in brain vessels is not associated with severity of disease

**Figure 3:**
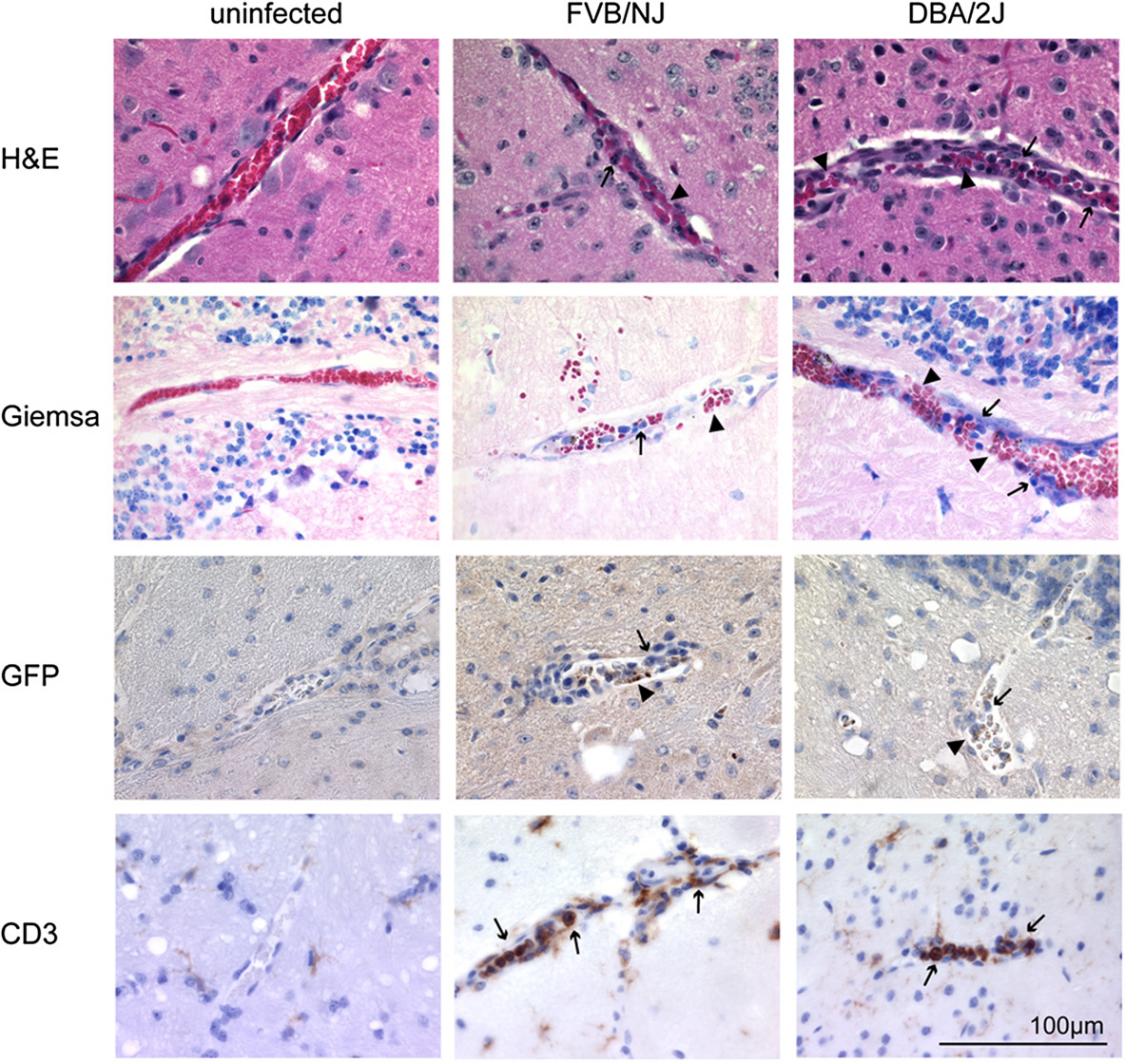
**Histological analysis of mouse brains.** Brains from three infected male FVB/NJ and DBA/2J, and from control mice were prepared for histological analysis on day eight post-infection. Shown are representative images for sagittal brain sections that were stained with Giemsa or E&H for general brain pathology (first two rows). Parasites used in the infection express GFP and were visualized with an anti-GFP antibody (third row) and lymphocytes with an anti-CD3 antibody (last row). Arrowheads indicate infected red blood cells and arrows indicate leukocytes.

As vascular leakage was found in brains of both strains, differences in the brains of susceptible and resistant mice was further investigated early during infection. Sequestration of mature parasites in peripheral tissue by cytoadherence of infected RBCs to the vascular endothelium is a common feature of *P. falciparum* malaria. Sequestration of parasites in the brain is observed in humans, as well as in mice, but its contribution to CM remains controversial (reviewed in [40]). Six brains of male mice on day eight, when FVB/NJ mice had started to show signs of ECM and the DBA/2J males had not, were examined. To more closely mimic the conditions found in human brains after autopsy, mice were not perfused prior to dissection. For general morphological differences brain sections were stained with H&E and Giemsa. The blood vessels contained infected RBCs and there was a marked increase in the amount of leukocytes present in brains from infected mice, compared to the controls (Figure 3). There was no obvious difference between the FVB/NJ and the DBA/2J mice.

The *P. berghei* strain used in this study carried a GFP protein under the control of an apical membrane associated 1 gene promoter that is expressed in schizonts and early rings [41]. Therefore, anti GFP antibodies were used to detect the parasite load in the brain. The staining of GFP confirmed the presence of parasites in the brains of infected mice from both strains (Figure 3). In addition to sequestration of parasites in the brain there is also an accumulation of lymphocytes (mainly CD8 T cells) in the brain of infected mice (reviewed in [42]). To specifically detect the amount of T cells in the brains, sections were stained with anti-CD3 antibodies. Although there were almost no T cells present in uninfected brains, brains from FVB/NJ and DAB/2-J mice showed massive infiltration (Figure 3). Anti-CD3 antibodies stained the majority of the nucleated cells and therefore most of the leukocytes observed in Giemsa and H&E stains were T cells. Again, no obvious difference in lymphocyte numbers between FVB/NJ and DBA/2J mice was found. Thus the presence of parasites and leukocytes (mainly T cells) in the brain is not correlated with CM or early death in FVB/NJ mice.

### High basophil counts in the peripheral blood are associated with resistance

**Figure 4:**
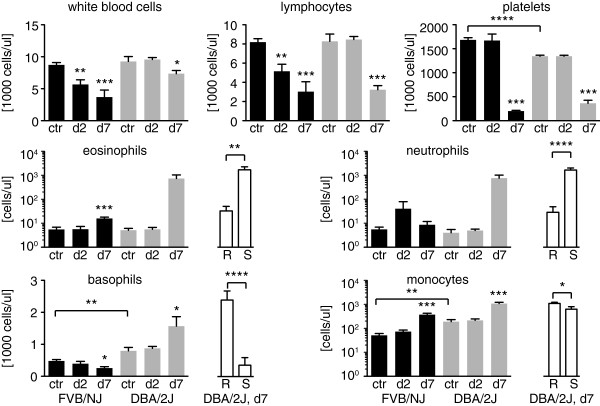
**Peripheral blood analysis.** Peripheral blood was taken from three control mice from each strain as well as from eight FVB/NJ mice (four males and four females) and from 20 DBA/2J mice (ten males and ten females) on day two and day seven post infection. Results for FVB/NJ mice are shown in black and in grey for DBA/2J mice. The white bars show significant differences between susceptible (S) and resistant (R) DBA/2J mice on day seven post infection. Shown are the averages and the standard errors. Statistically significant differences between control mice from different strains or between susceptible and resistant DBA/2J mice on day seven were calculated by unpaired Student’s t tests. Statistically significant differences between control mice and infected mice on day two and seven were calculated by one-way ANOVA with Dunnett’s post-hoc test, * p < 0.05, ** p < 0.01, *** p < 0.001, **** p < 0.0001.

Because no major differences were found in brains from mice that died early compared to mice that survived longer, the peripheral blood composition was examined in uninfected mice as well as infected mice on days two and seven post-infection. Significant differences between the uninfected parental control mice were observed: FVB/NJ control mice had significantly higher platelet counts than DBA/2J control mice and significantly lower basophil and monocyte counts than DBA/2J control mice (Figure 4).

Changes in the blood composition over the course of infection were observed for infected mice compared to their strain-specific uninfected control mice. Both strains showed a significant reduction in the number of platelets on day seven although the reduction was greater in FVB/NJ mice (8.5) than in DBA/2J mice (3.7). The total white blood cell (WBC) count also decreased significantly in both strains during the course of infection. FVB/NJ mice showed a significant decrease on day two whereas WBC counts did not drop significantly in DBA/2J mice until day seven. The same was true for lymphocytes, which account for the majority of WBC in control mice (94% in FVB/NJ and 89% in DBA/2J). This is in contrast to the accumulation of CD3 positive lymphocytes in the brain on day seven seen by histology. Sequestration of lymphocytes in the brain and other organs might be the reason for the reduction of lymphocytes in the peripheral blood.

As the DBA/2J mice consisted of those that were resistant (i.e. survived for over thirteen days) and susceptible (i.e. died before day thirteen), their peripheral blood composition was compared on day seven separately as well. Eosinophils increased significantly in FVB/NJ mice on day seven as well as in susceptible but not resistant DBA/2J mice (Figure 4). There was a significant negative correlation between eosinophil counts on day seven and survival in DBA/2J mice (r = -0.69, p = 0.0007). Although there was no change in neutrophil counts in FVB/NJ mice, susceptible DBA/2J mice showed a significant increase and neutrophil counts on day seven correlated inversely with survival for DBA/2J mice (r = -0.81, p > 0.00001). Monocytes increased significantly on day seven in both strains however the increase was significantly higher in resistant DBA/2J mice than in susceptible mice. The overall increase in monocytes was higher in FVB/NJ (7.2) than in DBA/2J (5.7) but this increase in monocytes did not seem to protect the mice from early death and there was only a weak correlation between monocyte counts on day seven and survival in DBA/2J mice (r = 0.55, p = 0.0114). Although basophil numbers on day seven decreased in FVB/NJ and susceptible DBA/2J mice they increased significantly in resistant DBA/2J mice. There was a strong correlation between basophil counts on day seven and survival in DBA/2J mice (r = 0.796, p < 0.0001). Susceptible mice of both strains behaved similar in the dynamics of WBC and platelets during the course of infection with the exception of basophils. While basophil counts on day seven dropped in susceptible mice from both strains, resistant mice showed an increase. Therefore, an increase in basophils on day seven was associated with protection from early death.

### Survival is linked to a locus on chromosome 9

**Figure 5:**
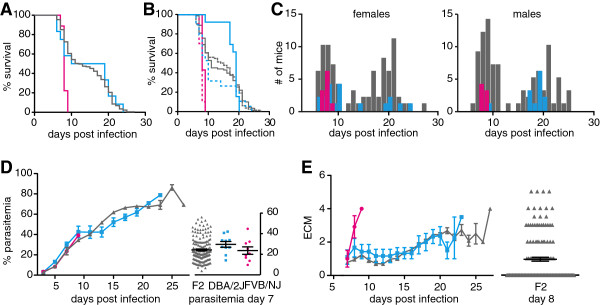
**Phenotypic analysis of F2 cross between DBA/2J and FVB/NJ.** 175 F2, twelve DBA/2J and nine FVB/NJ mice were infected and monitored twice daily. **A**. Survival for total DBA/2J (blue), FVB/NJ (pink) and F2 (grey) mice. **B**. Survival for male (solid lines) and female (dotted lines) mice. **C**. Distribution of survival for female (left) and male mice (right), colors as in **A**. **D**. Course of parasitaemia during infection and on day seven on the right. **E**. ECM scores over the course of infection with distribution of ECM scores for F2 mice on day eight to the right. Shown are the averages with the standard errors. Statistical analyses are discussed in the text.

To determine a genetic component that might be responsible for the early death of FVB/NJ mice and the bimodal survival distribution in DBA/2J, the F2 generation of a cross between the FVB/NJ mice and the DBA/2J mice was analysed. Parasitaemia, ECM scores and survival for 175 F2 mice as well as twelve DBA/2J and nine FVB/NJ control mice were recorded (Figure 5).

The survival distribution was also bimodal in the F2 generation, with a median survival of eight days for mice that died before day thirteen and a median of 20 days for mice that died after day thirteen. Survival was not linked to gender in the F2 generation (Figure 5A B and C) as there was no significant difference in survival between F2 males and females (p = 0.66). The linear slope of the parasitaemia of the F2 mice was not significantly different from that of the parents (Figure 5D, p before and after accounting for the baseline effect of gender was 0.44 and 0.53, respectively) and the terminal parasitaemia correlated also with survival (r = 0.796, p < 0.0001). The significant difference in parasitaemia seen previously between FVB/NJ and DBA/2J mice on day seven was not observed. The increase in F2 ECM scores was comparable to the DBA/2J parental mice with a steep increase in ECM in susceptible mice and a slower increase in resistant mice (Figure 5E). There was a borderline significant difference in the linear part of the slope of the ECM curve for the F2 mice compared to the parental mice (p = 0.08). The degree of ECM was higher in the F2 mice where 28% had ECM scores of more than 3.5 compared to their parents (10% in DBA/2J and 12.5% in FVB/NJ). 84% of these high ECM scores occurred in mice that died before day thirteen. Even though mice were checked three times daily, 48% of the F2, 73% of FVB/NJ and 85% of DBA/2J mice were found dead. It is therefore possible that a number of these mice would have progressed to severe disease and the terminal ECM scores might have been higher. Due to this uncertainty and the fact that there was no difference in parasitaemia, the survival trait was chosen as the most robust trait for quantitative trait locus analysis (QTL).

**Figure 6:**
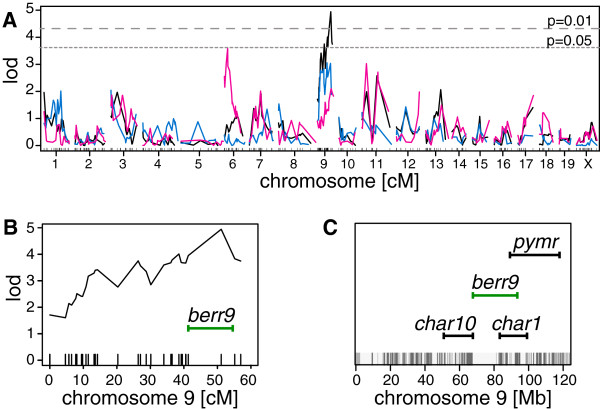
**Quantitative trait analysis for survival phenotype.** Quantitative trait locus analysis was performed on 149 F2 mice with 343 markers. **A**. Shown are the LOD scores plotted against 343 genetic markers on all chromosomes for all 149 F2 mice (black), for 80 males (blue) and 69 females (pink). The dotted lines indicate p value cut offs based on permutation testing of the data. **B**. Lod score profile for chromosome 9 and location of *berr9* (green). **C**. Positions of loci *char1*, *char10*, and *pymr* that are associated with control of malaria resistance on chromosome 9 [17,24,25,43]. Regions with genetic differences between FVB/NJ and DBA/2J mice are indicated as grey lines at the bottom of the graph (from Perlegen Mouse SNP Browser [44]).

To identify the QTL underlying the survival phenotype, all F2 mice were genotyped for 355 informative markers across all chromosomes. Quantitative trait analysis was performed in Rqtl across all chromosomes with survival as a quantitative trait. Only mice and markers with over 80% coverage were used (149 mice and 310 markers). This analysis identified a peak on chromosome 9. An additional 33 markers were used to refine this locus to a region consisting of 26.6 Mb between position 67.6 (rs6317714) and 94.2 Mb (rs13480351) on chromosome 9 (peak LOD score = 4.9, peak marker = rs13480311, Figure 6). This region, termed *berr9*, is adjacent to the *char10* (51.3 to 68.3 Mb) and overlaps the *char1* locus (83.9 to 114.8 Mb, Figure 6C), both regions have previously been identified by linkage studies with *P. chabaudi*[17,24,25]. It also overlaps with a locus called *Pymr,* (89.9 to 118.8 Mb) associated with resistance of mice to *Plasmodium yoelii* infections [43]. These findings strengthen the importance of this locus in the development of host resistance in murine malaria.

## Discussion

In depth characterization of FVB/NJ, a new mouse strain for malaria research, and DBA/2J, another strain used in malaria research, revealed similarities in the brain pathology between the strains but with differences in the peripheral blood composition. Mapping of the survival phenotype in a F2 cross identified a new locus on chromosome 9 that overlaps with a locus associated with parasite control in *P. chabaudi* (*char1*) and a locus identified in the *P. yoelii* parasitaemia model (*pymr*).

Rupture of the blood brain barrier and haemorrhage in the brain has been associated with susceptibility to ECM while being absent in ECM resistant mouse strains [45,46]. However, similar amounts of leakage of Evans blue into brains from resistant as well as susceptible mice were detected. In contrast, uninfected mice did not show leakage of Evans blue into the brain, suggesting that brains of infected mice differed in the permeability of the dye into the brain tissue and was not an artifact. In addition, parasites as well as CD3+ cells were found in the brain vasculature of resistant mice. This discrepancy between these studies and those of others might be due to methodological differences. In this study, mice were not perfused prior to brain preparation to better mimic the pathology observed in human brains. Therefore, the study detected not only parasites and CD3+ cells adhering to the endothelial walls but also those circulating in the brain vasculature. This might result in an overestimation of parasite and lymphocyte load in infected brains and might signify a difference in the number of parasites and lymphocytes actively sequestered in susceptible or resistant mice. Also, while the number of parasites and CD3+ cells in the brains of FVB/NJ and DBA/2J mice was comparable there could have been a difference in the state of inflammation between the strains.

In contrast to the brain pathology, several differences in the composition of the peripheral blood could be discriminated over the course of the infection between susceptible and resistant mice. A drop in circulating lymphocytes in the peripheral blood coincided with the accumulation of CD3+ cells in the brain of infected mice from both strains. While the frequency of these most common leukocytes decreased, less abundant leukocytes, such as monocytes, increased. An increase in circulating monocytes regardless of disease severity suggested that they play a minor role in disease outcome in this model. The only leukocytes that showed an inverse correlation between the susceptible and the resistant mice were basophils, where an increase correlated with resistance to early disease.

ECM pathogenesis in *P. berghei* infection is associated with a Th1 response involving TNF and IFN-γ cytokine secretion while a Th2 response characterized by IL-10 and TGF-β is protective (reviewed in [47]). The protective effect of IL-10 was demonstrated by the partial protection of susceptible mice from ECM upon administration of IL-10; in contrast, a neutralizing anti-IL-10 antibody induced ECM in a resistant strain [48]. Basophils are a central component in skewing a Th1 to a Th2 immune response by the release of IL-4 [49] which stimulates the generation of IL-10 producing CD8+ T cells [50]. The observed increase in basophil counts in the resistant DBA/2J mice might therefore indicate that the prevailing Th1 response shifted to a less harmful Th2 response, which allows the mice to survive for an additional week. Further studies will be needed to study the role of basophils in malaria infection.

Survival was the most robust resistance trait that distinguished the different mouse strains. While a resolving phenotype as observed previously in DBA/2J mice, could not be reproduced [38,45], a bimodal survival distribution was shown. A higher parasite dose used for infection might be a reason for this stronger phenotype observed here as mice have less time to mount a protective immune response. The survival distribution in the F2 offspring was also bimodal and mapping of the survival trait in the F2 cross of FVB/NJ and DBA/2J mice identified a locus on chromosome 9. This locus overlapped partially with *char1* and *pymr*, two loci previously identified using parasitaemia as resistance trait in crosses between two susceptible strains, SJL and C3H/He, and the resistant strain C57BL/6 in the *P. chabaudi* and a backcross of the susceptible NC/Jic and the resistant 129/SvJ in the *P. yoelii* model, respectively [17,43]. A region within *char1* has been further prioritized by a quantitative trait analysis of peak parasitaemia in a C57BL/6 J and SM/J cross [25]. However, this refined region does not overlap with the locus identified here. Even though a *P. berghei* model for ECM was used, the chromosome 9 locus overlaps with two loci identified in murine malaria models for resistance to the blood stage form of malaria. It is, therefore, possible that the response to the parasites in the blood is of greater importance than is the response to the parasites in the brain (in this particular strain combination). This is also in agreement with the fact that no major differences in brain pathologies was observed for DBA/2J and FVB/NJ mice, but instead differences were found in the peripheral blood composition.

While the markers chosen for the QTL analyses are in regions of high genetic variability between the two mouse strains, the sequences between 68 and 83 Mb on chromosome 9 contains almost no genetic markers that are different between FVB/NJ and DBA/2J mice (Figure 6C) and genes in this region are unlikely candidates for the phenotypic differences observed between the mouse strains.

## Conclusions

Mouse models are useful tools for the studies of ECM as well as for genetic analysis of susceptibility to malaria. The results of this study emphasize previously identified loci involved in susceptibility or resistance to malaria. Genes in this region, such as Rora, Irak1bp1, or Ibtk, might be good targets for intervention studies for CM. In addition, an association between basophils and resistance to malaria early in infection was shown and stimulating these cells might improve CM outcome in patients.

## Abbreviations

CM: Cerebral malaria; ECM: Experimental cerebral malaria; RBCs: Red blood cells.

## Competing interests

We declare that none of the investigators has any conflict of interest. None of the funders had any role in the evaluation, design, data collection, analysis, interpretation, drafting of the manuscript, or decision to publish.

## Authors’ contributions

SERB designed and executed the study, performed experiments, analysed the data, and drafted the manuscript. ER, MF and GEGP performed experiments. CV performed statistical analysis. SWB and JRW designed and performed the genotyping of the F2 mice. JW performed the histological analysis. CS and EAW provided material, reviewed and discussed the experimental data, and wrote the manuscript. All authors read and approved the final manuscript.

## References

[B1] Schofield L Grau GE Immunological processes in malaria pathogenesis Nat Rev Immunol 2005 5 722 10.1038/nri1686 16138104

[B2] Aikawa M Brown A Smith CD Tegoshi T Howard RJ Hasler TH Ito Y Perry G Collins WE Webster K A primate model for human cerebral malaria: *Plasmodium* coatneyi-infected rhesus monkeys Am J Trop Med Hyg 1992 46 391 397 1374220 10.4269/ajtmh.1992.46.391

[B3] Ibiwoye MO Howard CV Sibbons P Hasan M van Velzen D Cerebral malaria in the rhesus monkey (*Macaca mulatta*): observations on host pathology J Comp Pathol 1993 108 303 310 10.1016/S0021-9975(08)80293-9 8315058

[B4] Gysin J Aikawa M Tourneur N Tegoshi T Experimental *Plasmodium falciparum* cerebral malaria in the squirrel monkey *Saimiri sciureus* Exp Parasitol 1992 75 390 398 10.1016/0014-4894(92)90252-6 1493871

[B5] Curfs JH van der Meide PH Billiau A Meuwissen JH Eling WM *Plasmodium berghei*: recombinant interferon-gamma and the development of parasitemia and cerebral lesions in malaria-infected mice Exp Parasitol 1993 77 212 223 10.1006/expr.1993.1078 8375490

[B6] Rest JR Cerebral malaria in inbred mice. I. A new model and its pathology Trans R Soc Trop Med Hyg 1982 76 410 415 10.1016/0035-9203(82)90203-6 7051459

[B7] de Souza JB Riley EM Cerebral malaria: the contribution of studies in animal models to our understanding of immunopathogenesis Microbes Infect 2002 4 291 300 10.1016/S1286-4579(02)01541-1 11909739

[B8] Penet MF Viola A Confort-Gouny S Le Fur Y Duhamel G Kober F Ibarrola D Izquierdo M Coltel N Gharib B Grau GE Cozzone PJ Imaging experimental cerebral malaria *in vivo*: significant role of ischemic brain edema J Neurosci 2005 25 7352 7358 10.1523/JNEUROSCI.1002-05.2005 16093385 PMC6725296

[B9] Lackner P Beer R Helbok R Broessner G Engelhardt K Brenneis C Schmutzhard E Pfaller K Scanning electron microscopy of the neuropathology of murine cerebral malaria Malar J 2006 5 116 10.1186/1475-2875-5-116 17125519 PMC1676017

[B10] Wassmer SC Combes V Grau GE Pathophysiology of cerebral malaria: role of host cells in the modulation of cytoadhesion Ann N Y Acad Sci 2003 992 30 38 10.1111/j.1749-6632.2003.tb03135.x 12794044

[B11] von Zur MC Sibson NR Peter K Campbell SJ Wilainam P Grau GE Bode C Choudhury RP Anthony DC A contrast agent recognizing activated platelets reveals murine cerebral malaria pathology undetectable by conventional MRI J Clin Invest 2008 118 1198 1207 18274670 10.1172/JCI33314 PMC2242620

[B12] Grau GE Fajardo LF Piguet PF Allet B Lambert PH Vassalli P Tumor necrosis factor (cachectin) as an essential mediator in murine cerebral malaria Science 1987 237 1210 1212 10.1126/science.3306918 3306918

[B13] Pais TF Chatterjee S Brain macrophage activation in murine cerebral malaria precedes accumulation of leukocytes and CD8+ T cell proliferation J Neuroimmunol 2005 163 73 83 10.1016/j.jneuroim.2005.02.009 15885309

[B14] Grau GE Piguet PF Engers HD Louis JA Vassalli P Lambert PH L3T4+ T lymphocytes play a major role in the pathogenesis of murine cerebral malaria J Immunol 1986 137 2348 2354 3093572

[B15] Bopp SE Ramachandran V Henson K Luzader A Lindstrom M Spooner M Steffy BM Suzuki O Janse C Waters AP Zhou Y Wiltshire T Winzeler EA Genome wide analysis of inbred mouse lines identifies a locus containing Ppar-gamma as contributing to enhanced malaria survival PLoS ONE 2010 5 e10903 10.1371/journal.pone.0010903 20531941 PMC2878346

[B16] Driss A Hibbert JM Wilson NO Iqbal SA Adamkiewicz TV Stiles JK Genetic polymorphisms linked to susceptibility to malaria Malar J 2011 10 271 10.1186/1475-2875-10-271 21929748 PMC3184115

[B17] Foote SJ Burt RA Baldwin TM Presente A Roberts AW Laural YL Lew AM Marshall VM Mouse loci for malaria-induced mortality and the control of parasitaemia Nat Genet 1997 17 380 381 10.1038/ng1297-380 9398834

[B18] Fortin A Belouchi A Tam MF Cardon L Skamene E Stevenson MM Gros P Genetic control of blood parasitaemia in mouse malaria maps to chromosome 8 Nat Genet 1997 17 382 383 10.1038/ng1297-382 9398835

[B19] Lin E Pappenfuss T Tan RB Senyschyn D Bahlo M Speed TP Foote SJ Mapping of the *Plasmodium chabaudi* resistance locus char2 Infect Immun 2006 74 5814 5819 10.1128/IAI.01690-05 16988259 PMC1594909

[B20] Fortin A Cardon LR Tam M Skamene E Stevenson MM Gros P Identification of a new malaria susceptibility locus (Char4) in recombinant congenic strains of mice Proc Natl Acad Sci U S A 2001 98 10793 10798 10.1073/pnas.191288998 11535821 PMC58554

[B21] Hernandez-Valladares M Naessens J Gibson JP Musoke AJ Nagda S Rihet P Ole-MoiYoi OK Iraqi FA Confirmation and dissection of QTL controlling resistance to malaria in mice Mamm Genome 2004 15 390 398 10.1007/s00335-004-3042-4 15170228

[B22] Hernandez-Valladares M Rihet P Iraqi FA ole-MoiYoi OK Mapping of a new quantitative trait locus for resistance to malaria in mice by a comparative mapping approach with human Chromosome 5q31-q33 Immunogenetics 2004 56 115 117 10.1007/s00251-004-0667-0 15118851

[B23] Min-Oo G Fortin A Pitari G Tam M Stevenson MM Gros P Complex genetic control of susceptibility to malaria: positional cloning of the Char9 locus J Exp Med 2007 204 511 524 10.1084/jem.20061252 17312006 PMC2137903

[B24] Min-Oo G Willemetz A Tam M Canonne-Hergaux F Stevenson MM Gros P Mapping of Char10, a novel malaria susceptibility locus on mouse chromosome 9 Genes Immun 2010 11 113 123 10.1038/gene.2009.78 19865104

[B25] Laroque A Min-Oo G Tam M Radovanovic I Stevenson MM Gros P Genetic control of susceptibility to infection with *Plasmodium chabaudi chabaudi* AS in inbred mouse strains Genes Immun 2012 13 155 163 10.1038/gene.2011.67 21975430 PMC4912355

[B26] Bagot S Campino S Penha-Goncalves C Pied S Cazenave PA Holmberg D Identification of two cerebral malaria resistance loci using an inbred wild-derived mouse strain Proc Natl Acad Sci U S A 2002 99 9919 9923 10.1073/pnas.152215199 12114535 PMC126600

[B27] Nagayasu E Nagakura K Akaki M Tamiya G Makino S Nakano Y Kimura M Aikawa M Association of a determinant on mouse chromosome 18 with experimental severe *Plasmodium berghei* malaria Infect Immun 2002 70 512 516 10.1128/IAI.70.2.512-516.2002 11796577 PMC127666

[B28] Ohno T Nishimura M Detection of a new cerebral malaria susceptibility locus, using CBA mice Immunogenetics 2004 56 675 678 10.1007/s00251-004-0739-1 15536567

[B29] Campino S Bagot S Bergman ML Almeida P Sepulveda N Pied S Penha-Goncalves C Holmberg D Cazenave PA Genetic control of parasite clearance leads to resistance to *Plasmodium berghei* ANKA infection and confers immunity Genes Immun 2005 6 416 421 10.1038/sj.gene.6364219 15973462

[B30] Berghout J Min-Oo G Tam M Gauthier S Stevenson MM Gros P Identification of a novel cerebral malaria susceptibility locus (Berr5) on mouse chromosome 19 Genes Immun 2010 11 310 318 10.1038/gene.2009.79 19865103

[B31] Torre S van Bruggen R Kennedy JM Berghout J Bongfen SE Langat P Lathrop M Vidal SM Gros P Susceptibility to lethal cerebral malaria is regulated by epistatic interaction between chromosome 4 (Berr6) and chromosome 1 (Berr7) loci in mice Genes Immun 2013 14 249 257 10.1038/gene.2013.16 23594960

[B32] Gonçalves LA Almeida P Mota MM Penha-Gonçalves C Malaria liver stage susceptibility locus identified on mouse chromosome 17 by congenic mapping PLoS ONE 2008 3 e1874 10.1371/journal.pone.0001874 18365019 PMC2267218

[B33] Sepulveda N Paulino CD Carneiro J Penha-Goncalves C Allelic penetrance approach as a tool to model two-locus interaction in complex binary traits Heredity (Edinb) 2007 99 173 184 10.1038/sj.hdy.6800979 17551528

[B34] Fortin A Diez E Rochefort D Laroche L Malo D Rouleau GA Gros P Skamene E Recombinant congenic strains derived from A/J and C57BL/6J: a tool for genetic dissection of complex traits Genomics 2001 74 21 35 10.1006/geno.2001.6528 11374899

[B35] Wiltshire T Pletcher MT Batalov S Barnes SW Tarantino LM Cooke MP Wu H Smylie K Santrosyan A Copeland NG Jenkins NA Kalush F Maual RJ Flynne RJ Kay SA Adams MD Fletcher CF Genome-wide single-nucleotide polymorphism analysis defines haplotype patterns in mouse Proc Natl Acad Sci U S A 2003 100 3380 3385 10.1073/pnas.0130101100 12612341 PMC152301

[B36] Franke-Fayard B Trueman H Ramesar J Mendoza J van der Keur M van der Linden R Sinden RE Waters AP Janse CJ A *Plasmodium berghei* reference line that constitutively expresses GFP at a high level throughout the complete life cycle Mol Biochem Parasitol 2004 137 23 10.1016/j.molbiopara.2004.04.007 15279948

[B37] Broman KW Wu H Sen S Churchill GA R/qtl: QTL mapping in experimental crosses Bioinformatics 2003 19 889 890 10.1093/bioinformatics/btg112 12724300

[B38] Neill A Hunt N Pathology of fatal and resolving *Plasmodium berghei* cerebral malaria in mice Parasitology 1992 105 165 175 10.1017/S0031182000074072 1280805

[B39] Perlmann P Troye-Blomberg M rev. and enl Malaria immunology 2002; 2007 2 Switzerland: Karger

[B40] de Souza JB Hafalla JC Riley EM Couper KN Cerebral malaria: why experimental murine models are required to understand the pathogenesis of disease Parasitology 2010 137 755 772 10.1017/S0031182009991715 20028608

[B41] Franke-Fayard B Janse CJ Cunha-Rodrigues M Ramesar J Buscher P Que I Lowik C Voshol PJ den Boer MA van Duinen SG Febbraio M Mota MM Waters AP Murine malaria parasite sequestration: CD36 is the major receptor, but cerebral pathology is unlinked to sequestration Proc Natl Acad Sci U S A 2005 102 11468 11473 10.1073/pnas.0503386102 16051702 PMC1183563

[B42] Renia L Potter SM Mauduit M Rosa DS Kayibanda M Deschemin JC Snounou G Gruner AC Pathogenic T cells in cerebral malaria Int J Parasitol 2006 36 547 554 10.1016/j.ijpara.2006.02.007 16600241

[B43] Ohno T Ishih A Kohara Y Yonekawa H Terada M Nishimura M Chromosomal mapping of the host resistance locus to rodent malaria (*Plasmodium yoelii*) infection in mice Immunogenetics 2001 53 736 740 10.1007/s00251-001-0390-z 11862405

[B44] Perlegen Mouse SNP Browserhttp://mouse.cs.ucla.edu/perlegen/

[B45] Neill AL Chan-Ling T Hunt NH Comparisons between microvascular changes in cerebral and non-cerebral malaria in mice, using the retinal whole-mount technique Parasitology 1993 107 Pt 5 477 487 8295787 10.1017/s0031182000068050

[B46] Thumwood CM Hunt NH Clark IA Cowden WB Breakdown of the blood–brain barrier in murine cerebral malaria Parasitology 1988 96 Pt 3 579 589 2457201 10.1017/s0031182000080203

[B47] Hunt NH Grau GE Cytokines: accelerators and brakes in the pathogenesis of cerebral malaria Trends Immunol 2003 24 491 499 10.1016/S1471-4906(03)00229-1 12967673

[B48] Kossodo S Monso C Juillard P Velu T Goldman M Grau GE Interleukin-10 modulates susceptibility in experimental cerebral malaria Immunology 1997 91 536 540 10.1046/j.1365-2567.1997.00290.x 9378491 PMC1363872

[B49] Sokol CL Barton GM Farr AG Medzhitov R A mechanism for the initiation of allergen-induced T helper type 2 responses Nat Immunol 2008 9 310 318 10.1038/ni1558 18300366 PMC3888112

[B50] Kim S Shen T Min B Basophils can directly present or cross-present antigen to CD8 lymphocytes and alter CD8 T cell differentiation into IL-10-producing phenotypes J Immunol 2009 183 3033 3039 10.4049/jimmunol.0900332 19667092

